# Maternal Work–Life Balance and Children’s Social Adjustment: The Mediating Role of Perceived Stress and Parenting Practices

**DOI:** 10.3390/ijerph18136924

**Published:** 2021-06-28

**Authors:** Rikuya Hosokawa, Toshiki Katsura

**Affiliations:** 1 Department of Human Health Sciences, Graduate School of Medicine, Kyoto University, Kyoto 606-8507, Japan; 2 Faculty of Nursing, Meiji University of Integrative Medicine, Kyoto 629-0392, Japan; t_katsura@meiji-u.ac.jp

**Keywords:** mothers’ work–life balance, mothers’ perceived stress, mothers’ parenting practices, children’s social adjustment behavior

## Abstract

The participation of women with young children in the Japanese labor force has increased dramatically in recent years, bringing growing potential for conflict between work and family roles amid inadequate social systems, such as childcare support. Thus, work–life balance (WLB) of mothers may influence their children’s mental health and lifestyle. This study aims to clarify the relationship between parents’ WLB and children’s mental health, as well as the underlying factors of parental stress and nurturing attitude. The study is based on a questionnaire survey administered in 2019 to fifth-grade elementary school students and their caregivers in Aichi Prefecture, Japan. The regression results indicated that the higher the work–family negative spillover, the higher the child’s externalizing and internalizing problems, and the higher the positive spillover, the lower the problems and the higher the prosocial behaviors. Path analysis indicates that maternal WLB is negatively and positively related to children’s behavior through maternal stress and parenting practices. The study suggests that maternal WLB is related to children’s emotional and behavioral problems. WLB may impact children’s emotional and behavioral problems through parents’ mental health and involvement with their children, particularly because of work arrangements changing with the COVID-19 pandemic.

## 1. Introduction

Work–life balance (WLB), the balance between an individual’s work and family life, has attracted increasing research attention [1,2,3]. There is concern that work–family imbalance can lead to poor health and performance for individuals, families, and organizations. For most individuals, the most dominant areas of life are work and family. WLB is an individual’s perception that work and non-work activities are compatible and can promote growth in accordance with the individual’s current life priorities [4]. For families with children, WLB is understood to be satisfaction and good functioning, with minimal conflict between parental roles in the work and home domains [5]. Positive spillovers from work to home are associated with health and well-being. A good balance of work and life predicts good well-being and overall quality of life (QOL) [6]. However, work–family conflict is associated with lower life satisfaction and lower QOL; robust associations between depression, psychological distress, poor self-rated health, and unhealthy behaviors have been documented [7,8,9,10,11]. Parents raising children are a key subject in the WLB literature for understanding their family life and career trajectory. For many workers, both men and women, it is becoming increasingly important to balance work and family roles. Working parents experience not only struggles and hardships, but also rewards and joys, and the balance between the two can vary greatly depending on life course. Balancing work with childcare is easier in some situations than others. WLB is assessed as the degree to which work has both a positive and a negative impact on the family [12]. Negative spillovers have been studied extensively in the work–family literature [13]. However, to understand the work and family experiences of working parents across the life course, it is important to focus on all spillovers, including positive aspects, in a multidimensional way. However, few studies have examined both the positive and negative aspects of how parents’ WLB is related to their children’s emotional and behavioral problems.

The composition of the labor force worldwide has changed dramatically over the last few decades. The number of women in the labor force will continue to grow [14]. As a result of this trend, increasing numbers of workers are engaging in dual-earner lifestyles, where both partners in a family work and share family caregiving, and routinely take on some family responsibilities [15]. With the increase in dual-income households and changes in non-traditional gender roles, balancing work and family domains has become part of everyday life for many families. Some countries have a prevalence of single-parent households for various reasons. These households may experience similar issues. WLB affects both men and women, but in general, women bear the majority of family responsibilities, especially after childbirth [16]. Balancing work and home life for women after childbirth affects them in many ways [17,18]. Traditional attitudes toward women’s work and family roles persist in many Organisation for Economic Co-operation and Development (OECD) countries. On the one hand, on average, across OECD countries, about one-fourth of women believe that mothers with young children should not work because of childcare in the period after the birth of the child [19]. On the other hand, on average, more than half of the women in the OECD and the EU believe that mothers with young children should be allowed to work, mainly because of changing gender roles. Japan is also a society in which gender roles are changing but are still emphasized. Traditionally, men worked and women performed housework and cared for the children, but nowadays, women in Japan are increasingly participating in the workforce compared to the past [20]. In Japan, the number of dual-earner households is increasing, and now exceeds the number of single-earner households [20]. The number of women who quit their jobs because of pregnancy or childcare is on the decline, and the number of working mothers, many of whom continue to work while raising their children, is on the rise [20]. As women enter the workforce, the roles of women and men in the home have also changed [20]. Traditionally, society has regarded women as being responsible for housework and childcare in the home. Nowadays, however, there is widespread acknowledgment that both men and women should be involved in household chores and childcare. It has become increasingly important for both men and women to balance their work and family roles. In recent years, changes in the industrial structure and diversification of work styles have made it difficult to distinguish clearly between work time and non-work time, and WLB has become an important issue for workers. There have been concerns about the impact of working style and WLB of dual-earner couples on the lifestyle and mental health of their children. In Japan, mothers tend to be anxious about childcare, as fathers tend to participate less in childcare and mothers tend to take responsibility for the care of their children [21]. Japanese fathers work longer hours and participate in significantly less housework and childcare than do fathers in other OECD countries [19,20,21]. As a result, childcare is left to the mother, which leads to anxiety about childcare. The purpose of this study is to clarify the relationship between maternal WLB and their children’s risk of social maladjustment.

WLB can affect the mental health of parents, influence nurturing attitudes, and affect child development. Work–family conflict may be regarded as a stressor for individuals [22]. The COVID-19 pandemic has sped up rapidly changing work styles. The pandemic and other factors have increased the prevalence of a variety of work arrangements, including working from home. Flexible work arrangements have been found to be associated with lower mental health scores in the postpartum period, and may have other unintended consequences, such as increasing the amount of work taken home [16]. Research has established that conflict between work and home domains has several significant negative consequences for individuals. Work–life conflicts are associated with indicators of poor health and impaired well-being, such as higher levels of stress, increased levels of anxiety and depression, and fatigue [23]. Meanwhile, improved WLB fosters not only job satisfaction and job performance, but also family satisfaction; WLB also reduces stress-related outcomes, such as psychological distress, emotional exhaustion, anxiety, and depression [24]. Parenting stress may make optimal parenting more difficult and negatively affect child development [25]. Parenting-related stress can affect the family’s atmosphere and contribute to parenting dysfunction. Parents who report high levels of parenting stress are more likely to have authoritarian, harsh, and negative parenting practices, and are less engaged with their children [26]. Although the relationship between parenting-related stress, nurturing attitudes, and child development has been clarified, the relationship from WLB to stress, nurturing attitudes, and child development has not.

It is possible that maternal WLB influences children’s mental health and lifestyle. However, the relationship between maternal WLB and children’s mental health has not been sufficiently clarified. In addition, the role of stress and nurturing attitudes in the relationship between maternal WLB and children’s mental health, which underlie the relationship between maternal WLB and children’s mental health, is unknown.

Therefore, this study aims to clarify the relationship between maternal WLB and children’s mental health, as well as the relationship between maternal stress and nurturing attitude, which are considered intermediate factors.

## 2. Methods

### 2.1. Participants

In this study, a self-reported questionnaire was administered to fifth-grade students (10–11 years old) and their caregivers in 2019. The study used data from a portion of a larger study examining the childcare environment and child development. Participants were recruited for the overall study in 2014 in all nursery schools and kindergartens in the city of Nagoya, Aichi Prefecture, Japan. This study used data from a prospective cohort study that examined child development, adjustment, and influencing factors. Participants were recruited from 130 kindergartens and nursery schools with permission from the head of the institution. Parents provided written informed consent for their children to participate in the study. The study is conducted annually as the children grow and seeks to track their development from preschool through middle school. Baseline indicators were established by obtaining the addresses of participants in the first wave of the study conducted in 2014. The present study used data from the sixth wave conducted in 2019. In this wave, one questionnaire per child was distributed to the child’s parents. Parents then completed the questionnaires and returned them to the researchers by mail. Self-reported questionnaires were distributed to participants who were deemed suitable for follow-up, in this case, parents of 10- and 11-year-old children in the fifth grade of elementary school (*n* = 1414). Of the 1414 questionnaires distributed, 720 responses were obtained (response rate: 52.2%). This study examined the relationship between the WLB of working mothers and the development of their children. As described in the introduction, given that fathers tend to participate less in childcare than mothers, who tend to take responsibility for their children’s care, it can be assumed that mothers are more likely to have difficulties with WLB. Therefore, the purpose of this study was to clarify the relationship between maternal WLB and their children’s risk of social maladjustment. Therefore, among the 709 valid responses, we excluded children diagnosed with developmental disabilities, questionnaires answered by people other than mothers, and mothers who were not working. As a result, the total number of subjects analyzed in this study was 473.

### 2.2. Ethics Statement

Ethical approval for this study was obtained from the Ethics Committee of Kyoto University (E2322). All procedures performed in the study with human participants were in accordance with the standards of the Ethics Committee. Before participating in the study, parents provided written informed consent on behalf of their children.

### 2.3. Measures

#### 2.3.1. Work–Life Balance: Survey Work–Home Interaction—NijmeGen

The WLB of mothers was assessed using the Survey Work–Home Interaction—NijmeGen (SWING) [27]. This scale evaluates four aspects of WLB: (1) work–family negative spillover: 8 items; (2) family–work negative spillover: 4 items; (3) work–family positive spillover: 5 items; and (4) family–work positive spillover: 5 items. Respondents were asked to use a 4-point scale ranging from 0 (never) to 3 (always). The reliability and validity of the Japanese version have been confirmed previously, and we used the Japanese version in this study [28]. In this study, the internal consistency of the subscales was confirmed (Table S1). To clarify how the impact of WLB from work to home is related to family functioning and child development, in this study, we used two of the four subscales mentioned above: work–family negative spillover and work–family positive spillover.

#### 2.3.2. Mothers’ Perceived Stress: Perceived Stress Scale

Mothers’ stress was assessed using the Perceived Stress Scale (PSS) [29], which is one of the most widely used scales to measure the perception of stress. This scale measures the degree to which life situations are rated as stressful. The original version of the PSS consisted of 14 items (PSS-14), but this scale was later reduced to 10 items (PSS-10) [30]. The PSS-10 is recommended because it has more satisfactory psychometric properties than PSS-14 [31]. Items are rated on a 5-point scale ranging from 0 (never) to 4 (very often). The PSS-10 consists of six items that are positively expressed and four items that are negatively expressed. In this study, positive items were recorded as negative items. The higher the score, the higher the level of perceived stress. In Japan, the reliability and validity of the PSS-10 have been confirmed, and the present study is a Japanese version of the PSS-10 [32]. The internal consistency of the scale was confirmed in this study (Table S1).

#### 2.3.3. Mothers’ Parenting Practices: Alabama Parenting Questionnaire

Mothers’ parenting attitudes were measured using the Alabama Parenting Questionnaire (APQ) [33,34]. The APQ is a 42-item self-reported measure of parenting behavior with five subscales: (1) poor monitoring/supervision: 10 items; (2) inconsistent discipline: 6 items; (3) corporal punishment: 3 items; (4) positive parenting: 6 items; and (5) involvement: 10 items. Seven items in other discipline practices were not included in the subscale. The items were rated on a 5-point scale ranging from 1 (never) to 5 (always). The questionnaire had adequate internal consistency and construct validity. In this study, the internal consistency of the subscales was confirmed (Table S1). Furthermore, separate positive and negative parenting composite scores were standardized [35]. The negative parenting composite score was calculated by converting the poor monitoring/supervision, inconsistent discipline, and corporate punishment subscales to z-scores and averaging the z-scores. The higher the score, the higher the negative parenting. The positive parenting composite score was calculated in the same way as the positive parenting and involvement subscales, with higher scores indicating higher positive parenting.

#### 2.3.4. Child Behavior: Strengths and Difficulties Questionnaire

For children’s behavior, we used the Strengths and Difficulties Questionnaire (SDQ) for children [36,37]. The SDQ measures social competence and both extraverted and introverted behaviors. The SDQ is a 25-item child-behavior screening scale. Items are rated on a three-point scale ranging from 0 (not true) to 2 (certainly true). The SDQ consists of five subscales: emotional symptoms (5 items), behavioral problems (5 items), hyperactivity (5 items), peer problems (5 items), and prosocial behaviors (5 items). In the SDQ, the difficult behavior score is calculated by summing all scores, with higher scores indicating greater behavioral difficulties and a greater likelihood of having a mental disorder. However, for prosocial behaviors only, a higher score indicates greater social competence. On the basis of Goodman et al. [38], this study integrated the behavioral problems and hyperactivity/tension subscales into an externalizing problems scale, and integrated the subscales of peer problems and affective problems into an internalizing problem scale, all of which were analyzed. These scales showed satisfactory internal consistency. The reliability and validity of the Japanese version of the SDQ has been confirmed [39]. In the present study, the internal consistency of the subscales was confirmed (Table S1).

#### 2.3.5. Demographic Covariates

We collected self-reported information on children’s gender, family structure, household income, and parents’ education level. The gender of a child plays an important role in behavioral outcomes [40,41]. For example, previous studies have shown that boys tend to exhibit more externalizing problems than girls do. In the present study, the child’s gender was significantly associated with several child behavior variables (Table 1). Furthermore, family structure affects children’s behavior [42,43]. In the present study, family structure was significantly related to the behavioral variables of the children (Table 1). In addition, the socioeconomic status of the family may affect children’s behavior, including annual household income and parents’ education level. Certainly, children from disadvantaged families have greater behavioral problems [44,45]. In the present study, the socioeconomic status of the family was significantly associated with several children’s behavioral variables (Table 1). As a result, in the adjusted model of this study, we included the indicators of family socioeconomic status and child gender as covariates to account for these confounding factors.

### 2.4. Data Analyses

First, to examine the relationship between maternal WLB and children’s behavior, we conducted multiple regression analysis with maternal WLB as the independent variable and children’s behavior as the dependent variable.

The relationships between participants’ characteristics and their children’s behavior were analyzed using a *t*-test or one-way ANOVA (see Table 1). Although there were no significant relationships between some of the target attributes and children’s behavior, as mentioned above in Section 2.3.5, previous studies have shown relationships between gender, family socioeconomic status, and behavior. Thus, to maintain consistency of the analysis, we used gender, family structure, and socioeconomic status as adjustment variables in the analysis. Specifically, to examine the relationship between maternal WLB and children’s behavior, we conducted multiple regression analysis with WLB as the independent variable; children’s social maladaptive behavior as the objective variable; and gender, family structure, and household socioeconomic status as the adjustment variables (see Table 2). We evaluated multicollinearity between the predictors and found no problems.

**Figure 1:**
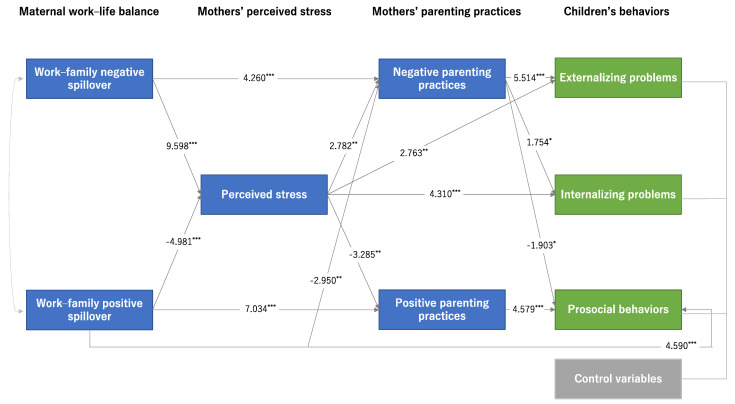
Path of maternal work–life balance, perceived stress, parenting practices, and children’s behaviors. Note: This model includes paths that are statistically significant in the hypothesized model. Control variables include children’s gender, family structure, family income, and parental educational attainment. Model fit statistics: χ^2^ (29) = 45.04; CFI = 0.98; IFI = 0.98; RMSEA = 0.03. * *p* < 0.05; ** *p* < 0. 01; *** *p* < 0.001.

Next, to explore the process of association between maternal WLB and children’s behavior, path analysis was conducted, including mothers’ stress and childcare attitudes. As a precursor to this analysis, correlations were conducted to measure the association between maternal WLB, maternal stress, parenting practices (negative and positive parenting practices), and child behavior (externalizing problems, internalizing problems, and prosocial behaviors) (see Table S2). Path analysis was then conducted to estimate the direct and indirect paths between maternal WLB, maternal stress, parenting practices, and child behavior (see Figure 1 and Table S3). Structural equation modeling analysis was conducted using full information maximum-likelihood estimation in the presence of missing data. A hypothetical model is shown in Figure S1. To evaluate the goodness of fit, we examined the comparative fit index (CFI), incremental fit index (IFI), and root mean square error of approximation (RMSEA) [46,47,48,49]. A good model fit is reflected in CFI and IFI values above 0.90. For RMSEA, goodness of fit is represented by values less than 0.05. All statistical analyses were performed using SPSS version 23.0 and Amos version 23.0.

## 3. Results

### 3.1. Participants’ and Children’s Behavior

Of the valid responses, 473 that met the inclusion criteria were included in the analysis. First, we examined the relationship between target attributes and children’s behavior (Table 1). In terms of gender, boys tended to have higher externalizing problems and lower prosocial behaviors than girls did. In terms of family structure, children from single-parent households tended to have higher internalizing problems than children from two-parent households. In terms of socioeconomic status (household income, mother’s education, and father’s education), children whose mothers had “compulsory education/upper secondary school” tended to have higher internalizing problems than those whose mothers had “up to 4 years at college/university” or “more than 4 years at college/university.”

### 3.2. Work–Life Balance and Children’s Behavior

#### 3.2.1. Work–Life Balance and Children’s Externalizing Problems

The relationship between maternal WLB and children’s externalizing problems is shown in Table 2. The relationship between maternal WLB and the child’s externalizing problems was analyzed using Model 1 (independent variables entered individually), Model 2 (all independent variables entered), and Model 3 (all independent variables and adjusted variables entered). In Models 1 and 2, the greater the negative influence from work to home, the higher the child’s externalizing problems tended to be; however, the greater the positive influence from work to home, the lower the child’s externalizing problems tended to be. Finally, in Model 3, the greater the negative impact from work to home, the higher the child’s externalizing problems (β = 0.123, *p* = 0.011), while the greater the positive impact from work to home, the lower the child’s externalizing problems (β = −0.161, *p* < 0.001).

#### 3.2.2. Work–Life Balance and Child’s Internalizing Problems

The relationship between maternal WLB and the child’s internalizing problems is shown in Table 2. In terms of the relationship between maternal WLB and child’s internalizing problems, Models 1 and 2 showed that the greater the negative impact from work to home, the higher the child’s internalizing problems tended to be; however, the greater the positive impact from work to home, the lower the child’s internalizing problems tended to be. Finally, in Model 3, the greater the negative impact from work to home, the higher the child’s internalizing problems (β = 0.164, *p* < 0.001); however, the greater the positive impact from work to home, the lower the child’s internalizing problems (β = −0.142, *p* = 0.003).

#### 3.2.3. Work–Life Balance and Child’s Prosocial Behaviors

The relationship between maternal WLB and child’s prosocial behaviors is shown in Table 2. Models 1 and 2 showed no significant relationship between the negative influence from work to home and prosocial behaviors; however, the greater the positive influence from work to home, the higher the child’s prosocial behaviors tended to be. Finally, in Model 3, there was no significant association between negative work–family influences and the child’s prosocial behaviors (β = −0.016, *p* = 0.726); however, the greater the positive work–family influence, the higher the child’s prosocial behaviors (β = 0.292, *p* < 0.001).

### 3.3. Path of Maternal Work–Life Balance, Perceived Stress, Parenting Practices, and Child Behaviors

Path analysis was conducted to estimate direct and indirect paths between maternal WLB, mothers’ stress, parenting practices (negative and positive), and children’s behavior (externalizing problems, internalizing problems, and prosocial behaviors) (see the hypothesized model in Figure S1). Figure 1 shows the final path model. The path diagram specifies the pathways that link maternal WLB to the child’s behavior (externalizing problems, internalizing problems, and prosocial behaviors). The standardization coefficients are shown in Figure 1 (see Table S3 for detailed results). The fit of the model was tested with several indices; the model provided a good fit to the data (χ^2^ (29) = 45.04, CFI = 0.98, IFI = 0.98, RMSEA = 0.03). In the model, several statistically significant direct and indirect paths were found between the predictors and the criterion variables. In terms of maternal WLB, work–family negative spillover increased perceived stress (β = 9.598, *p* < 0.001) and negative parenting practices (β = 4.260, *p* < 0.001). Work–family positive spillover decreased perceived stress (β = −4.981, *p* < 0.001), decreased negative parenting practices (β = −2.950, *p* < 0.001) and increased positive parenting practices (β = 7.034, *p* < 0.001). Furthermore, work–family positive spillover was directly related to prosocial behaviors (β = 4.590, *p* < 0.001). Next, perceived stress increased negative parenting practices (β = 2.782, *p* < 0.001) and decreased positive parenting practices (β = −3.285, *p* < 0.001). Furthermore, perceived stress was directly related to externalizing problems (β = 2.763, *p* < 0.001) and internalizing problems (β = 4.310, *p* < 0.001). Regarding mothers’ parenting practices, negative parenting practices increased externalizing problems (β = 5.514, *p* < 0.001), increased internalizing problems (β = 1.754, *p* < 0.001), and decreased prosocial behaviors (β = −1.903, *p* < 0.001). Positive parenting practices showed an increasing relationship with prosocial behaviors (β = 4.579, *p* < 0.001).

## 4. Discussion

The results of this study indicate that maternal WLB is related to children’s behavior in both negative and positive ways. Specifically, even after adjusting for children’s gender, family composition, family income, and parental educational attainment, we found that the higher the work–family negative spillover, the higher the child’s externalizing and internalizing problems. Meanwhile, the higher the work–family positive spillover, the lower the child’s externalizing and internalizing problems, and the higher the prosocial behaviors. Furthermore, path analysis was conducted to examine the direct and indirect paths between maternal WLB, mothers’ stress, parenting practices, and children’s behaviors. The results indicated that maternal WLB was related to children’s behavior both negatively and positively through the paths of maternal stress and parenting practices. Stress associated with WLB may be important for child behavior. These results are consistent with those of previous studies, as discussed below.

Regarding the association between WLB and perceived stress, working women with work–life imbalance may recognize that they bear a heavy burden. Conflict experienced by women between their work and family roles may be related to psychological distress [50]. An increase or decrease in WLB may affect the stress felt by mothers. To minimize the potential negative effects of stress, one must understand the mechanisms that influence parenting stress. One of these is the stress spillover process. Stress spillover occurs when stress that arises in response to difficulties within one area of a person’s life is sustained in other areas. External stressors have been consistently demonstrated to have a negative impact on family relationship satisfaction [51]. Regarding perceptions of parenting, stress spillover is thought to occur when stressful events or situations from outside the realm of parenting (e.g., problems at work and family disagreements) trigger negative emotional reactions while parents interact with or think about their children. Thus, impressions of the child and parenting in general are formed in the context of the parent’s negative emotional state and may be influenced by the parent’s emotions in a variety of ways.

Regarding the association between perceived stress and parenting practices, stressed parents tend to be more demanding and less likely to engage in positive parenting [52,53]. Parenting stress can make optimal parenting more difficult and can have a negative impact on children’s development [54]. Elevated negative emotions may increase a mother’s susceptibility to aversive stimuli, such as child distress [55]. Elevated negative emotions may promote negative perceptions of life in general, particularly unfavorable perceptions of parenthood [56]. Several findings support the application of the stress-spillover model in explaining the influence of contextual factors on parenting stress. Common symptoms of negative emotions, such as anxiety and depression, appear to influence mothers’ perceptions of their children. Studies have also reported the spillover effects of stress on other aspects of parenting, such as parental behavior and parent–child relationships [57]. To support positive child development, it is important to identify factors that may influence changes in parental behavior, of which one of the most promising is parental stress. Parental stress has been associated with less responsive, authoritarian, and neglectful parenting [58,59]. Increases or decreases in stress associated with WLB may be related to increases in children’s behavioral problems through ineffective parenting. In addition, stress is associated with poor parental mental health, marital conflict, and poor parental physical health [60,61,62]. Parents who report high levels of parenting stress are more likely to have negative parenting practices and are less engaged with their children [63]. Thus, stress may have a negative impact on the parenting environment, including factors other than nurturing attitudes.

Regarding the association between parenting practices and child behaviors, in the present study, negative parenting practices were positively associated with children’s externalizing and internalizing problems, and negatively associated with their prosocial behaviors. Previous studies have discussed the relationship between negative parenting practices and children’s externalizing and internalizing problems. Harsh punitive parenting and inconsistent parenting are commonly conceptualized in the research as “negative parenting,” because they are believed to have both short- and long-term negative effects on children [64]. Negative parental behaviors, such as overreacting, have been shown to be associated with children’s externalizing behavior problems [65,66]. Parental restrictions and punishments have been shown to be predictors of internalized problem behavior in children. One feature of the coercive process is the parent’s reaction to the child’s non-compliance. On the one hand, when a parent withdraws an order or fails to follow through, the child’s negative and resistant behavior is negatively reinforced. Although a parent’s withdrawal of a command or failure to follow through on a directive may relieve the parent by resolving the conflict, the result is temporary. On the other hand, the parent may resort to increasingly harsh parenting strategies in an attempt to control the child’s behavior. As this cycle continues, the rate and intensity of these behaviors increase significantly as the coercive responses are reinforced in the parent–child relationship.

Meanwhile, in the present study, positive parenting practices were positively associated with children’s prosocial behaviors. Previous studies discussing the relationship between positive parenting practices and children’s prosocial behaviors showed that positive parenting practices are expressed by the tendency of parents to show affection and support for their children, as well as to show concern and care for their needs [67]. Positive parenting means that parents are involved in their children’s lives, provide support, promote autonomy, and set rules. Positive behaviors, such as parental warmth and responsiveness, have been shown to contribute to the development of positive behaviors in children [68]. Parental warmth and involvement are thought to be related to the development of children’s prosocial behaviors in multiple ways. Parental warmth and involvement provide children with a sense of security, trust, and protection, and increase children’s sense of belonging and connectedness to others while decreasing children’s self-centeredness [69]. Nurturing parents model behaviors of emotional concern, caring, and comfort, which children are more likely to emulate [70]. In addition, warm and supportive parents are more likely to express positive emotions when interacting with their children; encourage children to express their emotions, both positive and negative; and ultimately provide opportunities to learn effective ways to improve self-regulation [71]. Thus, paying attention to children’s understanding of their own and others’ emotions and needs may support their emotional regulation and sensitivity [72].

In terms of the results of this study, it is possible that maternal WLB, through both negative and positive spillover, influences children’s behavior through the path of mother’s stress and parenting practices.

## 5. Limitations

The limitations of this study are as follows. First, this study was based on cross-sectional data, and it was not possible to analyze causal relationships. Further studies using longitudinal data are necessary in the future. Second, there was bias in the target population of this study. The subjects were limited to Aichi Prefecture, Japan, which might have led to bias. Third, this study did not include the perspective of fathers. In future research, it is important to include both mothers’ and fathers’ perspectives by including fathers’ WLB, stress, and childcare methods as variables. For comparison, and given the changing roles of families and the roles of both parents in families, it would make sense to include fathers in this discussion. Finally, we were not able to identify factors related to WLB (and negative and positive spillovers between work and family). This study used data from a portion of a survey examining the childcare environment and child development; the main data are focused on mothers and children, and there is less focus on fathers. Future research should include items that may be related to WLB, such as more hours worked, less available external childcare, specific occupations/industries, and fathers with higher hours worked.

## 6. Conclusions

The present study found an association between maternal WLB and children’s emotional and behavioral responses at age 10–11 years. The results suggest that WLB may impact children’s emotional and behavioral problems through their mothers’ mental health and involvement with their children. The results show that negative WLB is associated with stress and children’s behavior. Health measures/interventions to address negative WLB and improve maternal and child health outcomes should identify maternal stress related to WLB and build social systems/childcare support to improve and maintain maternal mental health. We contend that such an approach would improve child health outcomes.

In addition, the COVID-19 pandemic has sped up rapidly changing work styles. The pandemic and other factors have led to a variety of work arrangements, including working from home. Flexible work arrangements have been found to be associated with lower mental health scores in the postpartum period, and may have other unintended consequences, such as increasing the amount of work taken home [16]. We believe that it is important for children’s development to carefully examine the relationship between changes in work styles and children’s development, as well as to actively work to improve the WLB balance of mothers and fathers.

## Figures and Tables

**Table 1:** Participants’ and children’s behaviors.

			Externalizing Problems			Internalizing Problems			Prosocial Behaviors		
	*n*	%	M	SD	*p*	M	SD	*p*	M	SD	*p*
Gender											
Boy	226	47.8	5.26	3.21	<0.001	3.32	2.99	0.803	6.26	2.16	<0.001
Girl	247	52.2	4.03	2.94		3.26	2.62		7.06	2.00	
Family composition											
Single-parent household	30	6.3	4.71	2.87	0.863	4.56	3.20	0.015	6.30	2.40	0.336
Two-parent household	443	93.7	4.61	3.14		3.21	2.76		6.70	2.10	
Annual household income (JPY)											
<3,000,000	44	9.3	4.98	3.23	0.368	3.90	3.06	0.407	6.33	2.03	0.200
3,000,000–6,000,000	187	39.5	4.81	3.46		3.33	2.68		6.90	2.13	
6,000,000–9,000,000	139	29.4	4.64	2.99		3.34	2.95		6.54	2.17	
≥9,000,000	90	19.0	4.16	2.45		3.00	2.78		6.44	1.99	
Mother’s education level											
Compulsory education/upper secondary school	96	20.5	5.05	3.69	0.327	4.12	3.12	0.003	6.53	1.91	0.155
Up to 4 years at college/university	190	40.5	4.54	2.90		2.91	2.74		6.90	1.99	
More than 4 years at college/university	183	39.0	4.48	3.01		3.23	2.53		6.51	2.30	
Father’s education level											
Compulsory education/upper secondary school	120	26.8	4.81	3.07	0.327	3.44	2.84	0.649	6.91	2.08	0.354
Up to 4 years at college/university	69	15.4	5.00	3.51		3.05	2.75		6.66	1.97	
More than 4 years at college/university	259	57.8	4.44	3.10		3.24	2.78		6.57	2.14	

Abbreviations: Number (*n*), mean (M), standard deviation (SD), *p*-value (*p*).

**Table 2:** Work–life balance and children’s behaviors.

	Model 1					Model 2					Model 3				
	B	SE	β	*p*	Adjusted R^2^	B	SE	β	*p*	Adjusted R^2^	B	SE	β	*p*	Adjusted R^2^
Work–life balance and children’s externalizing problems															
Work–family negative spillover	0.102	0.035	0.136	0.004	0.016	0.096	0.035	0.128	0.006	0.038	0.093	0.036	0.123	0.011	0.060
Work–family positive spillover	−0.14	0.041	−0.16	<0.001	0.023	−0.138	0.041	−0.157	<0.001		−0.142	0.042	−0.161	<0.001	
Work–life balance and children’s internalizing problems															
Work–family negative spillover	0.108	0.031	0.163	<0.001	0.024	0.106	0.031	0.160	<0.001	0.048	0.109	0.032	0.164	<0.001	0.067
Work–family positive spillover	−0.127	0.036	−0.164	<0.001	0.025	−0.126	0.036	−0.161	<0.001		−0.11	0.037	−0.142	0.003	
Work–life balance and children’s prosocial behaviors															
Work–family negative spillover	−0.022	0.024	−0.044	0.356	0.001	−0.018	0.023	−0.036	0.423	0.085	−0.008	0.023	−0.016	0.726	0.115
Work–family positive spillover	0.172	0.026	0.294	<0.001	0.084	0.174	0.027	0.295	<0.001		0.171	0.027	0.292	<0.001	

Note: Model 1: independent variables are entered individually; Model 2: all independent variables (work–family negative spillover and work–family positive spillover) are entered; Model 3: all independent variables and adjusted variables (children’s gender, family composition, family income, and parental educational attainment) are entered. Abbreviations: unstandardized coefficient (B), confidence interval (CI), standard error (SE), standardized coefficient (β), *p*-value (*p*).

## Data Availability

Not applicable.

## Supplementary Materials

The following are available online at https://www.mdpi.com/article/10.3390/ijerph18136924/s1, Table S1. Descriptive statistics for the study variable. Table S2. Correlations between maternal work–life balance, perceived stress, parenting practices, and children’s behaviors. Table S3. Path analyses. Figure S1. Path of maternal work–life balance, perceived stress, parenting practices, and children’s behaviors.
