# Supplementary material for: Maternal Work–Life Balance and Children’s Social Adjustment: The Mediating Role of Perceived Stress and Parenting Practices
Source: Int J Environ Res Public Health. 2021 Jun 28;18(13):6924. doi: 10.3390/ijerph18136924 (PMC8297251; doi:10.3390/ijerph18136924)
Supplement: Supplementary file 1 [file ijerph-18-06924-s001.zip › ijerph-1195412-supplementary.pdf]

Table S1. Descriptive statistics for the study variables

Description	*Range*	*M*	*SD*	*α*
Work–Life Balance: Survey Work–Home Interaction–NijmeGen (SWING)				
Work–family negative spillover	0-24	4.96	4.26	0.87
Work–family positive spillover	0-15	6.96	3.62	0.86
Perceived Stress: Perceived Stress Scale (PSS)				
Perceived stress	0-40	18.72	6.06	0.82
Parenting Practices: Alabama Parenting Questionnaire (APQ)				
Poor monitoring/supervision	10–50	16.22	4.67	0.82
Inconsistent discipline	6–30	12.16	3.19	0.74
Corporal punishment	3–15	5.68	2.04	0.69
Positive parenting	6–30	22.51	3.82	0.81
Involvement	10–50	37.89	5.31	0.82
Children's Behaviors: Strengths and Difficulties Questionnaire (SDQ)				
Externalizing problems	0–20	4.62	3.13	0.76
Internalizing problems	0–20	3.29	2.80	0.68
Prosocial behaviors	0-10	6.68	2.11	0.71

Table S2. Correlations between maternal work–life balance, perceived stress, parenting practices, and children's behaviors

Variable	*1*	*2*	*3*	*4*	*5*	*6*	*7*	*8*
Maternal Work–Life Balance								
1. Work–family negative spillover	—							
2. Work–family positive spillover	-0.013	—						
Mothers' Perceived Stress								
3. Perceived stress	0.405***	-0.211***	—					
Mothers' Parenting Practices								
4. Negative parenting practices	0.271***	-0.171**	0.246***	—				
5. Positive parenting practices	-0.119*	0.351***	-0.245***	-0.344***	—			
Children's Behaviors								
6. Externalizing problems	0.136**	-0.160**	0.255***	0.340***	-0.225***	—		
7. Internalizing problems	0.163**	-0.164**	0.292***	0.195***	-0.194***	0.401***	—	
8. Prosocial behaviors	-0.044	0.294***	-0.061	-0.213***	0.331***	-0.290***	-0.137**	—

Table S3. Path analyses

Construct			*B*	*SE*	*β*	*p*
Maternal Work–Life Balance						
Work–family negative spillover	→	Perceived stress	0.405	0.042	9.598	<0.001
Work–family negative spillover	→	Negative parenting practices	0.240	0.056	4.260	<0.001
Work–family negative spillover	→	Positive parenting practices	-0.050	0.048	-1.039	0.299
Work–family negative spillover	→	Externalizing problems	-0.036	0.049	-0.739	0.460
Work–family negative spillover	→	Internalizing problems	0.032	0.047	0.677	0.498
Work–family negative spillover	→	Prosocial behaviors	0.009	0.048	0.195	0.845
Work–family positive spillover	→	Perceived stress	-0.217	0.044	-4.981	<0.001
Work–family positive spillover	→	Negative parenting practices	-0.160	0.054	-2.950	0.003
Work–family positive spillover	→	Positive parenting practices	0.325	0.046	7.034	<0.001
Work–family positive spillover	→	Externalizing problems	-0.059	0.048	-1.223	0.221
Work–family positive spillover	→	Internalizing problems	-0.067	0.047	-1.432	0.152
Work–family positive spillover	→	Prosocial behaviors	0.215	0.047	4.590	<0.001
Mothers' Perceived Stress						
Perceived stress	→	Negative parenting practices	0.161	0.058	2.782	0.005
Perceived stress	→	Positive parenting practices	-0.161	0.049	-3.285	0.001
Perceived stress	→	Externalizing problems	0.137	0.049	2.763	0.006
Perceived stress	→	Internalizing problems	0.205	0.048	4.310	<0.001
Perceived stress	→	Prosocial behaviors	0.070	0.048	1.463	0.144
Mothers' Parenting Practices						
Negative parenting practices	→	Externalizing problems	0.298	0.054	5.514	<0.001
Negative parenting practices	→	Internalizing problems	0.094	0.053	1.754	0.049
Negative parenting practices	→	Prosocial behaviors	-0.102	0.054	-1.903	0.047
Positive parenting practices	→	Externalizing problems	-0.062	0.050	-1.243	0.214
Positive parenting practices	→	Internalizing problems	-0.065	0.048	-1.345	0.178
Positive parenting practices	→	Prosocial behaviors	0.221	0.048	4.579	<0.001

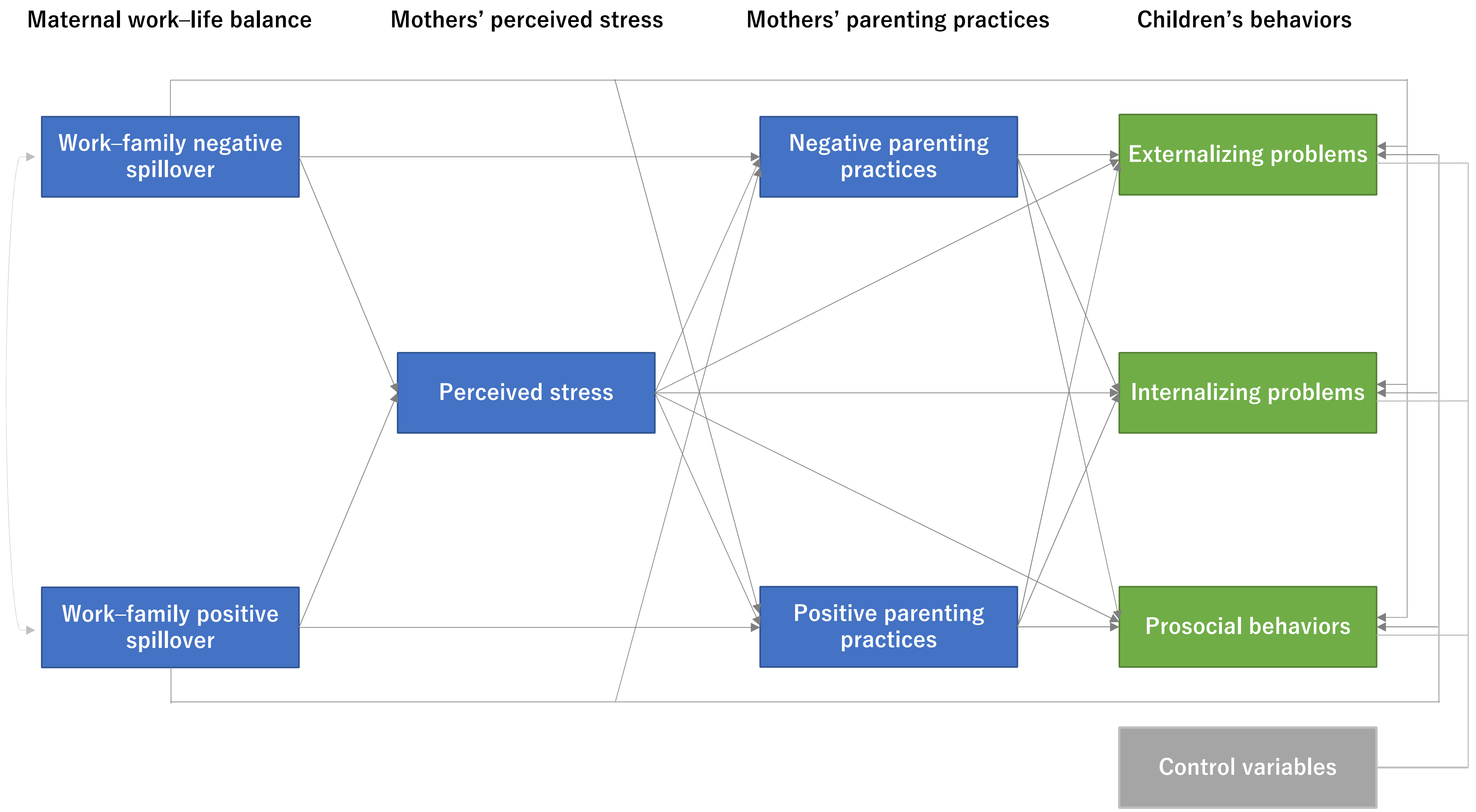

Figure S1. Path of maternal work–life balance, perceived stress, parenting practices, and children's behaviors

Note: This model includes the hypothesized pathways between maternal work–life balance, perceived stress, parenting practices, and children's behaviors.
